# Amlodipine-Induced Liver Injury

**DOI:** 10.7759/cureus.23441

**Published:** 2022-03-24

**Authors:** Julian Yet Kwong Horman, Puja Patel, Michael Schultz, Jennifer Kraschnewski

**Affiliations:** 1 Internal Medicine - Pediatrics, Penn State Health Milton S. Hershey Medical Center, Hershey, USA; 2 Internal Medicine, Penn State Health Milton S. Hershey Medical Center, Hershey, USA

**Keywords:** hypertension, idiosyncratic liver injury, drug induced liver injury, amlodipine liver injury, amlodipine

## Abstract

This is a case of an 88-year-old female with a history of hypertension who was started on amlodipine about three weeks prior to presentation. After about two weeks of amlodipine therapy, she developed intermittent right upper quadrant pain as well as pruritus which continued for a few days before she presented to medical attention. Her labs showed significantly elevated liver enzymes so she presented to the hospital for further evaluation. Imaging was unremarkable, her infectious and autoimmune workups were all negative. The amlodipine was discontinued and her liver enzymes slowly normalized after about seven weeks.

## Introduction

Amlodipine is a dihydropyridine calcium channel blocker that blocks the voltage-dependent L-type calcium channels. The blocking of these channels inhibits the influx of calcium into the cell, which lowers intracellular calcium causing decreased smooth muscle contractility leading to increased vasodilation. Amlodipine is approved for the treatment of hypertension, as well as chronic stable angina, vasospastic angina, and coronary artery disease [1]. The most recent hypertension guidelines recommend calcium channel blockers as a potential first-line medication for the treatment of hypertension [2]. Therefore, amlodipine is one of the most frequently prescribed medications in the United States. In 2018, amlodipine was the seventh most frequently prescribed medication in the United States and the second most frequently prescribed antihypertensive medication, only behind lisinopril [3]. 

Amlodipine is usually well tolerated with the most common side effect being peripheral edema [4]. Drug-induced liver injury is not a common side effect of amlodipine with only a handful of published reports in the literature. The incidence of drug-induced liver injury due to all medications is estimated to be around 14-19 per 1,00,000 patients although the incidence of drug-induced liver injury secondary to amlodipine is unknown [5].

Drug-induced liver injury can be classified based on the mechanism of injury, the pattern of injury, or histological findings. The mechanism of liver injury is further subdivided into intrinsic or idiosyncratic types. With an intrinsic injury, this refers to drugs that are known to cause liver injury in a predictable, dose-dependent fashion. Idiosyncratic drug-induced liver injury is less common, has a varied presentation, and has no obvious relation to dose [6].

## Case presentation

This is a case of an 88-year-old Caucasian woman with a history of osteoporosis who presented to the hospital at the behest of her primary care physician due to worsening transaminase elevation. About three weeks prior to presenting to the hospital she was diagnosed with hypertension and started on amlodipine. After about two weeks of amlodipine therapy, she started to have right upper quadrant abdominal pain and diffuse pruritus so she presented to her primary care physician where labs were drawn which returned with an alanine transaminase (ALT) of 876 U/L (normal value is less than 33 U/L), aspartate transaminase (AST) of 814 U/L (normal value is less than 32 U/L), alkaline phosphatase (ALK) of 167 U/L (normal range of 35-115 U/L), and total serum bilirubin (TSB) of 1.1 mg/dL (normal value is less than 1.2 mg/dL). The calculated R factor was 18.3 which is consistent with a hepatocellular pattern of injury. She was instructed to discontinue the amlodipine and to follow up in three days.

At her follow-up appointment, her labs were repeated and showed further elevation of the ALT and AST, increasing to 1247 and 1032 U/L, respectively. The ALK increased to 234 U/L as did the TSB, which increased to 2.5 mg/dL. The direct bilirubin was also elevated to 1.6 mg/dL (normal is less than 0.3 mg/dL). Due to the continued elevation of the liver enzymes, she was instructed to present to the hospital for further workup of her acute liver injury.

The patient underwent an extensive workup once at the hospital to evaluate the cause of her acute liver injury. Her infectious workup was negative and included Epstein-Barr, hepatitis A, hepatitis B, and hepatitis C viruses. The acetaminophen level was normal. Further evaluation revealed a negative antinuclear antibody (ANA), anti-mitochondrial antibody (AMA), liver-kidney-microsomal antibody, immunoglobulin G subclasses, and smooth muscle antibody. Her urine drug screen was also negative. An ultrasound of the right upper quadrant with duplex showed hepatic steatosis with mild liver nodularity at the gallbladder fossa as well as cholelithiasis without cholecystitis. There was no evidence of thrombosis in any of the vessels of the right upper quadrant. A CT scan of the abdomen was unremarkable and did not show any intrahepatic or extrahepatic biliary dilation.

Her liver function was closely monitored throughout her hospitalization (Table 1).

**Table 1:** Transaminase, alkaline phosphatase, and total serum bilirubin trend. The patient was admitted on day 4 and discharged on day 7.

Labs	Day 1	Day 4	Day 5	Day 6	Day 7	Day 11	Day 25	Day 53	Day 182
Alanine transferase	876 U/L	1247 U/L	1246 U/L	1100 U/L	890 U/L	651 U/L	125 U/L	27 U/L	12 U/L
Aspartate transferase	814 U/L	1032 U/L	963 U/L	858 U/L	623 U/L	473 U/L	103 U/L	40 U/L	28 U/L
Alkaline phosphatase	167 U/L	234 U/L	243 U/L	229 U/L	225 U/L	266 U/L	231 U/L	196 U/L	101 U/L
Total serum bilirubin	1.1 mg/dL	2.5 mg/dL	3.0 mg/dL	3.1 mg/dL	3.0 mg/dL	2.7 mg/dL	2.0 mg/dL	0.9 mg/dL	0.5 mg/dL

The ALT and AST slowly decreased during her hospitalization. On the day of discharge, her ALT was 890 U/L and her AST was 623 U/L. The TSB peaked at 3.1 mg/dL and was 3.0 mg/dL on the day of discharge. The ALK peaked at 243 U/L and was 225 U/L. The international normalized ratio (INR), hemoglobin, electrolytes, and renal function were monitored throughout the hospitalization and remained normal.

**Figure 1:**
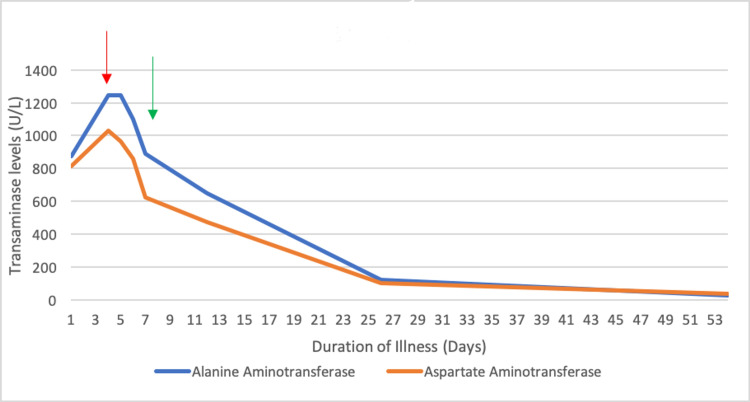
Transaminase trend. Red arrow indicating day of admission; green arrow indicating day of discharge.

She was discharged with close primary care physician follow-up. Her labs normalized seven weeks after discharge from the hospital, with the exception of the ALK which continued to be mildly elevated but normalized on subsequent evaluation (Figure 1).

## Discussion

Amlodipine is an uncommon cause of idiosyncratic drug-induced liver injury. The exact mechanism by which amlodipine causes injury is not known.

The R factor is a tool that can be helpful to differentiate hepatocellular, cholestatic and mixed liver injuries. The calculation is (patient's ALT/upper limit of normal ALT)/(patient's ALK/upper limit of normal ALK). Values greater than five are consistent with hepatocellular injury, values less than two are consistent with cholestatic injury, and values two to five are consistent with a mixed pattern of injury [7]. Calculating the R factor can be helpful to guide workup and treatment of the liver injury. The initial laboratory values for this woman showed significant elevation in the transaminases with mildly elevated ALK and normal TSB. Her R factor was significantly elevated, consistent with a hepatocellular pattern of injury. This is contrary to the liver injury that has been described with amlodipine, which is usually mild and asymptomatic with only mild elevations in the transaminases. The high calculated R factor is unusual to find in such cases as the pattern of injury seen with amlodipine is usually cholestatic or a mixed pattern of injury [8]. There are a few isolated case reports of amlodipine-induced hepatocellular injury. In all of these cases, the discontinuation of amlodipine resulted in a marked reduction of the transaminases with weeks.

Drug-induced liver injury is a diagnosis of exclusion and liver biopsy is not always required for diagnosis. Liver biopsy is often most useful to assess for alternative diagnoses. The European Association for the Study of the Liver (EASL) recommends liver biopsy during the initial evaluation for drug-induced liver injury if autoimmune hepatitis remains a competing etiology. Liver biopsy should also be considered if the suspected drug-induced liver injury progresses or does not improve after withdrawal of the suspected drug [9]. In this case, the antinuclear antibody (ANA) and AMA were negative, so the concern for autoimmune hepatitis was low, therefore liver biopsy was not performed. Furthermore, her transaminases improved after the cessation of amlodipine and had normalized after about seven weeks. When the liver biopsy is performed, the histologic findings of drug-induced hepatocellular injury usually show marked hepatocyte necrosis and inflammation with only mild bile stasis [10].

The cornerstone of treatment for idiosyncratic drug-induced liver injury is the discontinuation of the offending drug. The American College of Gastroenterology recommends N-acetylcysteine administration for treatment of early-stage idiosyncratic acute liver failure but does not recommend N-acetylcysteine in the treatment of drug-induced hepatitis [11]. As the patient, in this case, did not have any evidence of acute liver failure, N-acetylcysteine was not administered.

## Conclusions

Amlodipine is a commonly prescribed medication for the treatment of hypertension and is usually well tolerated. Many medications are known to cause drug-induced liver injuries, although the incidence differs depending on the specific drug. Amlodipine is a medication that can cause drug-induced liver injury, although this is a rare complication. The diagnosis of idiosyncratic drug-induced liver injury can be difficult due to the unpredictable presentation of the liver injury but prompt identification and cessation of the causative drug remain the cornerstone of treatment.
